# Recent Progress in Electrochemical Immunosensors

**DOI:** 10.3390/bios11100360

**Published:** 2021-09-29

**Authors:** JeeYoung Kim, Min Park

**Affiliations:** 1 Major in Materials Science and Engineering, Hallym University, Chuncheon 24252, Gangwon-do, Korea; jyoung@hallym.ac.kr; 2 Cooperative Course of Nano-Medical Device Engineering, Hallym University, Chuncheon 24252, Gangwon-do, Korea; 3 Integrative Materials Research Institute, Hallym University, Chuncheon 24252, Gangwon-do, Korea

**Keywords:** immunosensors, electrochemical immunosensors, biosensors, voltammetric immunosensors, amperometric immunosensors, impedimetric immunosensors, eletrochemiluminescent immunosensors

## Abstract

Biosensors used for medical diagnosis work by analyzing physiological fluids. Antibodies have been frequently used as molecular recognition molecules for the specific binding of target analytes from complex biological solutions. Electrochemistry has been introduced for the measurement of quantitative signals from transducer-bound analytes for many reasons, including good sensitivity. Recently, numerous electrochemical immunosensors have been developed and various strategies have been proposed to detect biomarkers. In this paper, the recent progress in electrochemical immunosensors is reviewed. In particular, we focused on the immobilization methods using antibodies for voltammetric, amperometric, impedimetric, and electrochemiluminescent immunosensors.

## 1. Introduction

A biosensor is an analytical device that can specifically quantify the target analyte in a physiological sample, such as blood, serum, plasma, cerebrospinal fluid, urine, and interstitial fluid [1,2]. Biosensors have the advantages of portability (because they are miniature), simplicity, automation, cost-effectiveness, high stability, and a short detection time [3,4,5,6,7]. Moreover, biosensors can provide real-time responses, and consequently, they are suitable for point-of-care testing [8]. Biosensors are used in various fields, including clinical diagnosis, agriculture, the food industry, environmental monitoring, and quality control [9].

Biosensors are normally composed of three main parts: a molecular recognition layer, a transducer, and a signal generator [2,10]. The molecular recognition layer of biosensors is distinct from that of other sensors because the sample analysis generally involves a complicated mixture in biosensors. The molecular recognition layer is produced by the immobilization of bioreceptors that have specific binding properties to target analytes [11,12]. Antibodies are one of the most widely used molecules because of their high specificity, affinity, and ease of production; therefore, they have been utilized in various applications such as chromatography, therapy, diagnosis, immunoassays, and biosensors [13,14]. Antigen–antibody binding-based biosensors are called immunosensors, and the antibody layers are called immunoaffinity layers.

There are various types of immunosensors depending on the transducer type, such as thermal, optical, magnetic, piezoelectric, fluorescent, and electrochemical biosensors [15,16]. Electrochemical biosensors measure electrochemical signals using a chemically modified electrode [17] and they have the distinct advantage of simplicity [18]. As described previously, electrochemical biosensors can be produced by the fabrication of chemically modified electrodes. Owing to their simplicity, electrochemical biosensors have the characteristics of good durability, easy miniaturization, small analyte volumes, and the ability to be integrated with fluidic systems. Another key advantage of electrochemical biosensors is their broad detection range and excellent detection limits [18]. Therefore, electrochemical biosensors, including electrochemical immunosensors, have been actively studied. 

In this review, various types of electrochemical immunosensors are introduced and discussed. In particular, the current information on voltammetric, amperometric, impedimetric, and ECL immunosensors is summarized (abbreviations are described in Table S1). This review focused on electrochemical immunosensors, and recent studies are discussed and summarized. In particular, the formation of an immunoaffinity layers for the fabrication of the electrochemical immunosensors is discussed. Recently, a couple of reviews focused on electrochemical biosensors have been published. Li et al. especially focused on CMOS and summarized the instrumentation of CMOS biosensors [19]. Juska and Pemble summarized the evolution of the electrochemical biosensor [20]. While this review focused on enzymatic electrochemical biosensors, especially glucose biosensors, our review focuses on antibody-based biosensors. Zhang and Yang focused on materials and techniques for the fabrication of electrochemical biosensors [21]. They summarized materials used for the electrode, immobilizing molecules, covering membrane and immobilization of molecular recognition molecules, and discussed various construction techniques for the preparation of biosensors. Schmidt-Speicher and Länge summarized electrochemical biosensors with integrated microfluidics [22]. The difference from the reviews discussed above is that our review focuses on immunosensors employing antibody immobilization techniques in the transducer.

## 2. Voltammetric Immunosensors

In voltammetric immunosensors, the current is obtained from the analyte measurements when the potential is changed. The potential between the working and reference electrodes changes over time, and the current generated between the working and counter electrodes vi the electrochemical reaction of the analytes is measured [23]. The signal originates from the oxidoreduction processes at the surface of the working electrode, caused by the electroactive species. According to the function of the applied potential, voltammetry is classified as LSV, DPV, SWV, or CV [24,25,26,27].

In voltammetric biosensors, the signal is normally collected from the peak or plateau, and the amount of the target analyte can be quantified as a proportion of the height of the peak. Voltammetry is an electrochemical analytical method for immunosensors that is widely used owing to its minimal noise, good sensitivity, and applicability [28,29]. In addition, voltammetry can be utilized for multiplex detection based on the different positions of the oxidoreduction peaks. The immune reaction cannot generate a voltammetric signal, so signal-generating molecules are essential for the fabrication of voltammetric immunosensors. From this reason, one of the major challenges in the development of voltammetric biosensors is the synthesis of highly sensitive signal-generating or -amplifying composites for the immunoaffinity layer or detection antibodies. In this section, voltammetric immunosensors employing various antibody immobilization methods are discussed, and examples are presented.

### 2.1. Physical Adsorption of Antibodies

**Figure 1:**
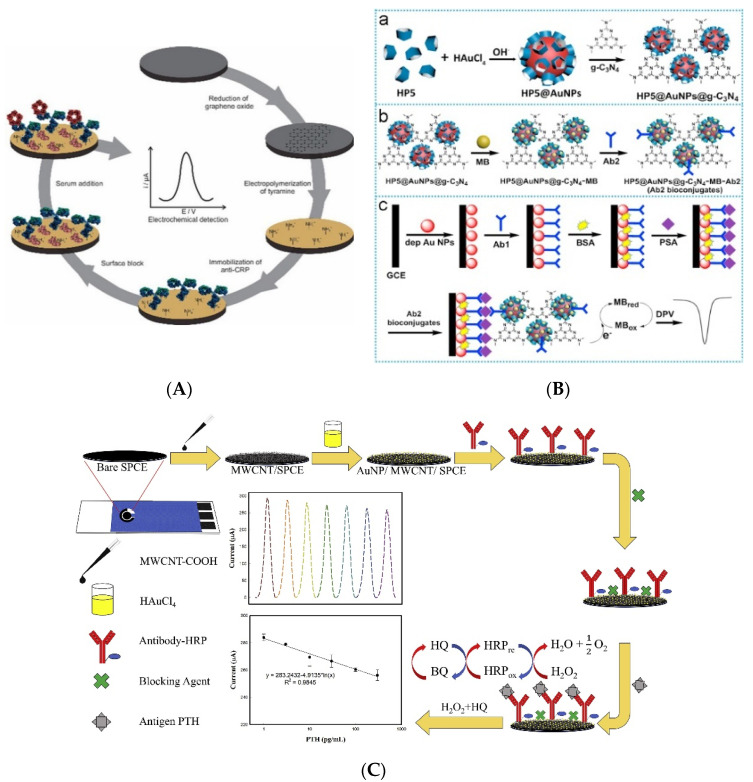
Examples of voltammetric immunosensors with non-covalently immobilized antibodies. (**A**) Schematic diagram of CRP immunosensor. Antibodies were immobilized on the polymer-modified electrode by the charge interaction. Reproduced with permission from [30]. Copyright (2020) John Wiley and Sons., Inc. (**B**) Immunosensor with non-covalently immobilized antibodies on AuNPs. Reproduced with permission from [33]. Copyright (2018) Elsevier. (**C**) Schematic diagram of immunosensor using gold and carbon-based materials at the same time. Reproduced with permission from [41]. Copyright (2021) Elsevier.

The formation of an immunoaffinity layer during the fabrication of electrochemical immunosensors, especially the immobilization of antibodies on the electrode, is an important process. Carbon-based materials are among the most widely used materials for electrode modification. Ribeiro et al. developed a reusable immunosensor by immobilizing antibodies on carbon-modified electrodes through non-covalent interactions and utilized them to detect CRP [30]. As shown in Figure 1A, the graphite electrode was first modified with graphene by electrochemical reduction using GO. Then, the electrode was coated with electropolymerized polytyramine. Antibodies against CRP were immobilized on the electrode through the charge interaction between the ammonium ion of polytyramine and the carboxyl group of the antibodies. After treatment with the analyte, CRP, the signal was measured using DPV. The fabricated immunosensor showed an LOQ of 1.25 µg/L, with a linear range of 1.09–100 µg/L, and it could be reused four times. Trindade et al. fabricated a label-free immunosensor for CysC detection [31]. The gold electrode was modified using an AF-functionalized GO nanocomposite, and then it was coated with PEI. Subsequently, antibodies against CysC were immobilized by simple drop casting. After the immunoaffinity layer was formed, CysC was quantified using SWV. From the measurement, the LOD and linear range were calculated to be 0.03 ng/mL and 0.1–1000 ng/mL, respectively.

Gold is another widely used material for electrode modification and antibody immobilization because of its biocompatibility [32]. Zhou et al. developed an electrochemical immunosensor based on antibody-immobilized AuNPs [33]. The transducer used in that study was composed of antibodies immobilized on the AuNP layer for the detection of PSA. HP5-decorated AuNPs were used for signal amplification. As shown in Figure 1B, the hybrid HP5–AuNP was modified by coating it with graphitic carbon nitride through π−π interactions between HP5 and graphitic carbon nitride. After the addition of methylene blue, the detection antibodies were conjugated with the nanocomposite through physical adsorption. The detection antibody conjugate was added after antigen treatment to form a sandwich-type immunocomplex, and the amplified signal was measured using DPV. The fabricated biosensor showed a 0.12 pg/mL LOD with a linear range of 0.0005–10.00 ng/mL. Suresh et al. utilized enzyme-conjugated detection antibodies for signal amplification to detect PSA [34]. AuNP was electrochemically synthesized on a CS-coated electrode, and capture antibodies were immobilized on the electrode through physical adsorption. After analyte treatment, HRP-labeled detection antibodies were added, and the signal was amplified by the reduction of hydrogen peroxide by HRP. The signal was measured using SWV, and the LOD and linear range of the developed immunosensor were calculated to be 0.001 ng/mL and 1–18 ng/mL, respectively. Zhang et al. similarly developed a PSA immunosensor based on a sandwich immunocomplex [35] and they prepared an immunoaffinity layer by sequentially coating the electrode with PANI, AuNPs, and antibodies. Then, methylene blue was encapsulated in mesoporous silica NPs, which were coated with PDA, and then detection antibodies were conjugated to the prepared particles. After the sandwich immunocomplex was formed, acid was added to release the encapsulated methylene blue, and the released methylene blue increased the signal of SWV. The fabricated immunosensor showed an LOD of 1.25 fg/mL with a linear range of 0.01–100 ng/mL. Li et al. fabricated a dual-mode immunosensor to detect procalcitonin, where antibodies were immobilized on a simple electrolytic gold matrix [36]. For signal generation, CuCo_2_O_4_ hollow spheres were coated with AuNPs, and detection antibodies were immobilized on the spheres. After the sandwich immunocomplex was formed, the signal was measured using dual mode, SWV and chronoamperometry, and the LODs of the developed immunosensor were calculated to be 82.6 fg/mL for SWV and 95.4 fg/mL for chronoamperometry, with 0.0001–50 ng/mL as the linear range. 

Various studies have utilized carbon-based materials together with gold. Amani et al. developed an immunosensor utilizing graphene and AuNPs for the detection of NSE [37]. The electrode was sequentially coated with graphene, PPD, and AuNPs. Subsequently, the antibodies were immobilized on the AuNPs through physical adsorption. After NSE treatment, the signal was measured using DPV, and the fabricated immunosensor showed an LOD of 0.3 ng/mL and a linear range of 1.0–1000 ng/mL. Khoshroo et al. fabricated a CA15-3 biosensor based on a cobalt sulfide-graphene nanocomposite [38]. In that study, AuNPs were coated on a CoS_2_–graphene modified electrode, and antibodies were immobilized on the AuNPs. CA15-3 was quantified using DPV, and the LOD and linear range were calculated to be 0.03 U/mL and 0.1 150.0 U/mL, respectively. Zhao et al. introduced platinum into an immunosensor to detect NMP22 [39]. They fabricated MOFs using AuNPs and PtNPs, and these MOFs were coated on a graphene-modified electrode. After NMP22 treatment, the signal was measured using DPV, and the fabricated immunosensor showed an LOD of 1.7 pg/mL with a linear range of 0.005–20 ng/mL. Assari et al. used CNTs to develop immunosensors for the detection of PSA [40]. They sequentially coated electrodes with MWCNTs, PANI, and AuNPs. Then, capture antibodies were immobilized on the modified electrode, and the signal measured with the immunosensor using DPV showed a calculated LOD and linear range of 0.5 pg/mL and 1.66 ag/mL–1.3 ng/mL, respectively. Chen et al. fabricated biosensors utilizing MWCNTs and AuNPs to detect PTH [41]. After sequentially coating the MWCNTs and AuNPs, antibodies against PTH were immobilized on AuNPs, and PTH was quantified using DPV and SWV (Figure 1C). The fabricated immunosensor showed an LOD of 886 fg/mL and 86 fg/mL for DPV and SWV, respectively, whereas the linear range was 1–300 pg/mL.

### 2.2. Chemical Immobilization of Antibodies

The chemical immobilization of antibodies using primary bonds has the advantage of providing robust, stable, and irreversible immobilization [2,42,43], which has led to its widespread use for the fabrication of immunoaffinity layers [42]. Shamsipur et al. developed an immunosensor based on the cross-linking of antibodies with silanized magnetite (Fe_3_O_4_) NPs to detect HER2 [43]. Magnetite NPs were functionalized with amine groups by treatment with APTMS, and antibodies were immobilized on the NPs by cross-linking with glutaraldehyde. Antibody-conjugated particles were immobilized on the electrode. The fabricated biosensor showed an LOD of 2.0 × 10^−5^ ng/mL with a linear range of 5.0 × 10^−4^–50.0 ng/mL, based on the DPV measurement.

**Figure 2:**
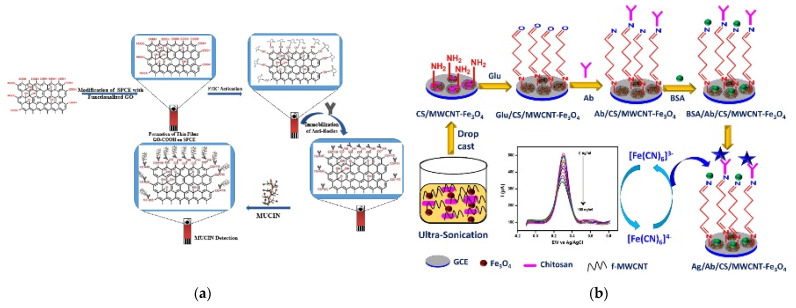
Examples of voltammetric immunosensors with covalently immobilized antibodies. (**a**) Immunosensor based on carboxyl functionalized GO. Antibodies were chemically immobilized using EDC chemistry. Reproduced with permission from [44]. Copyright (2018) Elsevier. (**b**) CA19-9 biosensor based on CS-MWCNT-Fe_3_O_4_. Antibodies were immobilized using glutaraldehyde. Reproduced with permission from [46]. Copyright (2021) Elsevier.

Similar to non-covalent immobilization, carbon-based materials have been widely used to chemically immobilize immunoaffinity layers. Rauf et al. utilized carboxyl-functionalized GO (GO–COOH) to fabricate a Mucin1 immunosensor [44]. As shown in Figure 2a, GO–COOH was assembled on the electrode and antibodies were immobilized using EDC. After Mucin1 was bound to the immunoaffinity layer, the signal was measured using DPV, and the LOD and linear range were calculated to be 0.04 U/mL and 0.1–50 U/mL, respectively. Devi et al. developed a microfluidic CysC immunosensor based on a CS–GO nanocomposite [45]. The electrode was modified using a nanocomposite and the antibodies against CysC were immobilized using EDC/NHS chemistry. The fabricated biosensor showed an LOD of 0.0078 mg/mL with a detection range of 1–10 mg/mL. Kalyani et al. fabricated a bio-nanocomposite using MWCNTs and magnetite NPs in CS, and used it to develop a CA19-9 immunosensor [46]. After depositing the bio-nanocomposite on the electrode, the antibodies were chemically immobilized using glutaraldehyde cross-linking (Figure 2b). The LOD and linear range were calculated to be 0.163 pg/mL and 0.001–100 ng/mL, respectively, based on SWV measurements. 

### 2.3. Other Methods

In the above section, immunosensors with antibodies immobilized using physical adsorption or chemical bonds were introduced. Unlike the studies described above, Sun et al. developed a voltammetric HSP70 immunosensor based on antigen immobilization [47]. They coated porous graphene onto the electrode and HSP70 was immobilized using physical adsorption. Then, the analyte HSP70 was pre-treated with biotinylated antibodies for a competitive assay. After the binding of the antibodies, streptavidin-conjugated HRP was added to generate the signal, which was measured using DPV. The fabricated immunosensor showed an LOD of 0.02 ng/mL with a linear range of 0.0448–100 ng/mL. Song et al. utilized affinity binding for the immobilization of antibodies to fabricate an electrochemical sensing system [48]. They immobilized avidin onto the ITO electrode using physical adsorption, and biotinylated capture antibodies were immobilized by biotin–avidin affinity binding. For signal generation, ALP-labeled detection antibodies were added after analyte binding. The fabricated immunosensor was tested to detect MMP-9 and Apo-A4 based on CV measurements. The developed immunosensor showed LODs of 0.21 and 6.6 ng/mL, with detection ranges 0.4–100 and 10–100 ng/mL for MMP-9 and Apo-A4 detection, respectively.

## 3. Amperometric Immunosensors

In amperometric immunosensors, the density or magnitude of the current is determined by measuring the electrochemical reactions at a constant voltage [9]. This technique has similar biosensing characteristics to those of other methods, such as the response time, dynamic range, and sensitivity [49,50]. In amperometry, reducing or oxidizing potential is generally applied to the working electrode, and the concentration of the reduced or oxidized substances is proportional to the measured current [51]. Similar to voltammetry, amperometry is one of the most widely used electrochemical analytical methods for immunosensors owing to its high selectivity. A high selectivity is achieved because the potential applied in the amperometric method is the specific reducing or oxidizing potential of the target [52]. In addition, amperometric biosensors require a minimal amount of analyte, so amperometric biosensors are suitable for monitoring analytes. Similar to the voltammetric immunosensors, a signal generation molecule is required in amperometric immunosensors, so the development of highly sensitive signal-generating or -amplifying composites is a key challenge for the fabrication of amperometric immunosensors.

### 3.1. Physical Adsorption of Antibodies

**Figure 3:**
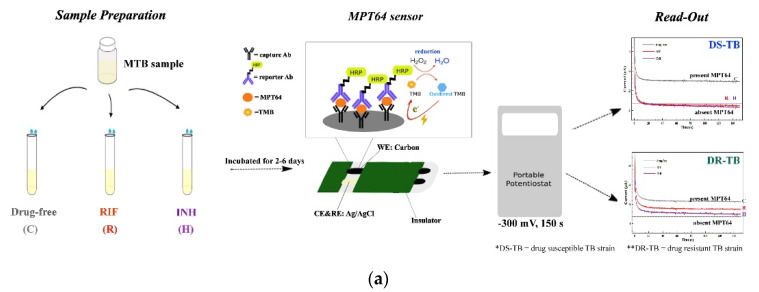

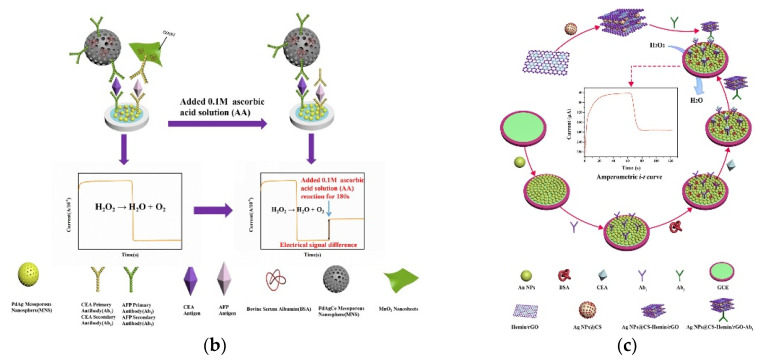
Examples of amperometric immunosensors with non-covalently immobilized antibodies. (**a**) Sandwich amperometric immunosensor. The immunoaffinity layer was fabricated by the direct treatment of antibodies on the electrode. Reproduced with permission [53]. Copyright (2018) Elsevier. (**b**) Composites-based immunosensor. Antibodies were immobilized on the PdAg nanosphere-modified electrode by physical adsorption. Reproduced with permission [55]. Copyright (2020) Elsevier. (**c**) AuNP-based immunosensor. An immunoaffinity layer was formed by immobilizing antibodies on the AuNP-coated electrode. Reproduced with permission [57]. Copyright (2020) Elsevier.

The physical adsorption of antibodies is also one of the most frequently used techniques for forming immunoaffinity layers. Chutichetpong et al. fabricated a disposable immunosensor based on a sandwich immunocomplex to detect MPT64 [53]. Capture antibodies were physically adsorbed onto the electrode to form an immunoaffinity layer, and HRP-conjugated detection antibodies were applied after antigen binding (Figure 3a). The signal was generated using the TMB reaction with HRP and measured using chronoamperometry. The fabricated immunosensor showed an LOD of 0.43 ng/mL, with two linear ranges of 0.3–50 ng/mL and 50–1000 ng/mL with different slopes.

Various metal composite-based sensors have been studied for the fabrication of amperometric immunosensors. Yan et al. developed a label-free amperometric biosensor for HBsAg detection [54]. In that study, AuPdCu ternary NPs were hydrothermally synthesized on N-GQDs, and the synthesized NPs were immobilized on PEI spheres using electrostatic attraction. The fabricated nanocomposites were immobilized on the electrode, whereas anti-HBsAg was immobilized using physical adsorption. After antigen binding, the signal was measured using amperometry, and the LOD and linear range were calculated to be 3.3 fg/mL and 10 fg/mL–50 ng/mL, respectively. Li et al. fabricated an amperometric immunosensor based on PdAg mesoporous nanospheres for the detection of multiple tumor markers [55]. As shown in Figure 3b, PdAg nanospheres were immobilized on the electrode and two antibodies against CEA and AFP were immobilized using physical adsorption. For signal generation, two types of detection antibodies were used: anti-CEA antibodies conjugated with PdAgCeO_2_ mesoporous nanospheres and anti-AFP antibodies labeled with manganese dioxide (MnO_2_) nanosheets. After sandwich immunocomplex formation, the first signal from the CEA and AFP was measured using amperometry. Then, the MnO_2_ nanosheet was eliminated using acid treatment, and the second signal was measured. The concentrations of AFP and CES antigens calculated from the signal difference between the two measurements were 0.001 ng/mL and 0.0005 ng/mL, with linear ranges of 0.005–100 ng/mL and 0.001–40 ng/mL, respectively.

Gold nanomaterials have also been widely used as amperometric immunosensors. Zhang et al. developed an AFP biosensor based on antibodies physically adsorbed on AuNPs [56]. AuNPs were coated on the electrode using electrodeposition and anti-AFP antibodies were immobilized. For signal amplification, PDA-functionalized phenolic resin microporous carbon spheres were decorated with silver-coated NPs for detecting antibody conjugation. The signal generated by the reduction of H_2_O_2_ was measured using amperometry, and the LOD and linear range were calculated to be 6.7 fg/mL and 20fg/mL–100 ng/mL, respectively. Zhang et al. fabricated an immunoaffinity layer using AuNPs for the detection of CEA [57]. As shown in Figure 3c, an immunoaffinity layer was formed by immobilizing antibodies on the AuNP-coated electrode, and then microporous carbon spheres loaded with AgNP-spaced Hemin/rGO porous composite materials were used as a label for the detection antibodies. This composite improves the catalytic activity by reducing H_2_O_2_; thus, the amperometric signal was amplified by composite detection antibody-binding. The developed immunosensor showed an LOD of 6.7 fg/mL with a linear range of 20–200 ng/mL. Yola et al. developed an amperometric galectin-3 immunosensor [58]. In that study, the capture antibody was conjugated with AuNP-functionalized g-C_3_N_4_, and a Ti-based MOF coated with a covalent organic framework was used as a label for the detection antibody for signal amplification. After forming the sandwich immunocomplex, the amperometric signal was measured and the results showed LOQ and LOD values of 0.10 pg/mL and 0.025 pg/mL, respectively, with a 0.1 pg/mL–20 ng/mL linear range. Yan et al. fabricated immunosensors using AuNRs and GO to detect HE4Ag [59]. They conjugated titanium oxide nanocluster-functionalized nitrogen-doped rGO with Pd-functionalized AuNR (AuNR@Pd) using silanization and modified electrodes with conjugated composites. The antibodies against HE4 were then immobilized using physical adsorption. The developed immunosensor showed an LOD of 13.33 fM and a 40–60 nM linear range.

### 3.2. Chemical Immobilization of Antibodies

**Figure 4:**
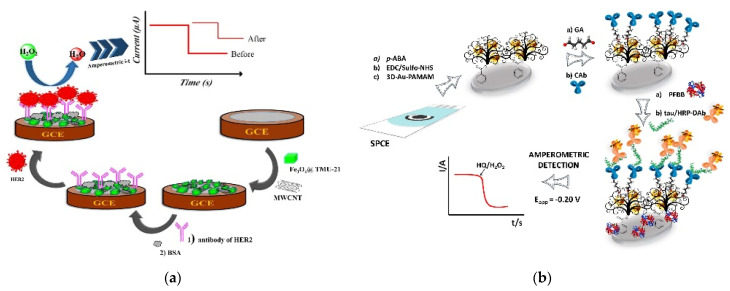
Examples of amperometric immunosensors with covalently immobilized antibodies. (**a**) Magnetite nanoparticles and MWCNT-based immunosensor. Antibodies were chemically immobilized with carboxylated MWCNTs through EDC/NHS chemistry. Reproduced with permission [61]. Copyright (2020) Elsevier. (**b**) AuNP-based immunosensor. Antibodies were immobilized on the amine group of the PAuNP-AMAM dendrimer nanocomposite using glutaraldehyde cross-linking. Reproduced with permission [63]. Copyright (2020) Elsevier.

Various studies have used chemically immobilized antibodies for amperometric biosensors. Martínez-Periñán et al. fabricated an endoglin immunosensor based on chemically immobilized antibodies [60]. For effective antibody immobilization, the electrode was modified with pPPA using electropolymerization to obtain an abundance of carboxyl groups. Then, the capture antibodies were immobilized onto the carboxyl group using EDC/NHS. After the analyte was bound, biotinylated detection antibodies and poly-HRP-conjugated streptavidin were sequentially added to amplify the signal. This biosensor showed an LOD of 140 pg/mL with a linear range of 0.1–600 ng/mL. Ehzari et al. developed an amperometric immunosensor using magnetite NPs and MWCNTs to detect HER2 [61]. The electrode was sequentially modified with TMU-21-decorated Fe_3_O_4_ (Fe_3_O_4_@TMU-21) and carboxylated MWCNTs, and the capture antibodies were immobilized through EDC/NHS chemistry (Figure 4a). The LOD and linear range of the fabricated amperometric immunosensor were 0.3 pg/mL and 1.0 pg/mL–100 ng/mL, respectively.

AuNPs are also widely used for the chemical immobilization of antibodies. Hou et al. immobilized capture antibodies using EDC/NHS chemistry on AuNPs to detect EV71 [62]. The electrode was modified with AuNPs using electrochemical deposition, and the AuNPs were modified with carboxyl groups by SAMs. Next, antibodies against EV71 were chemically immobilized onto the electrode. Dual-labeled magnetic nanobeads with antibodies and HRP were used for signal amplification, and the signal from the TMB reaction was measured using amperometry. The fabricated immunosensor showed an LOD of 0.01 ng/mL with a 0.1–600 ng/mL linear range. Razzino et al. also used AuNPs to develop an amperometric immunosensor for the detection of tau protein [63]. As shown in Figure 4b, the AuNP-PAMAM dendrimer nanocomposite was chemically immobilized on the electrode. Capture antibodies were immobilized on the maximum amine group of the PAMAM dendrimer using glutaraldehyde cross-linking. The signal was amplified using HRP-labeled detection antibodies and the amperometric measurement showed an LOD of 1.7 pg/mL with a 6–5000 pg/mL linear range.

## 4. Impedimetric Immunosensors

Electrochemical EIS is an electrochemical analytical method that analyzes combined resistive and capacitive properties [51]. EIS measures electron and mass transfer by scanning alternating current frequencies and quantifies dielectric properties, including resistive and capacitive properties and impedance [64]. Impedance is usually expressed as a complex number consisting of a real component (ohmic resistance) and an imaginary component (capacitive reactance), and Nyquist and Bode plots are generally used to analyze electrochemical impedance data [65,66,67]. In comparison with the voltammetry and amperometry, an impedance biosensor has the strong advantage of not requiring labeling [68]. This means that impedimetric immunosensors can detect the binding of an analyte without any additional signal generation or amplifying molecules. In addition, impedance analysis is highly sensitive with a high degree of accurate responses, as well as being stable and reproducible. Therefore, impedance has been used in both biosensors and various other applications, such as for clinical diagnosis, food analysis, environmental monitoring, and battery analysis [69,70,71]. For impedimetric immunosensors, capturing antibodies that form an immunoaffinity layer is one of the most important factors; therefore, effective antibody immobilization on the surface of the electrode is directly linked to improved biosensor performance [72].

### 4.1. Physical Adsorption of Antibodies

**Figure 5:**
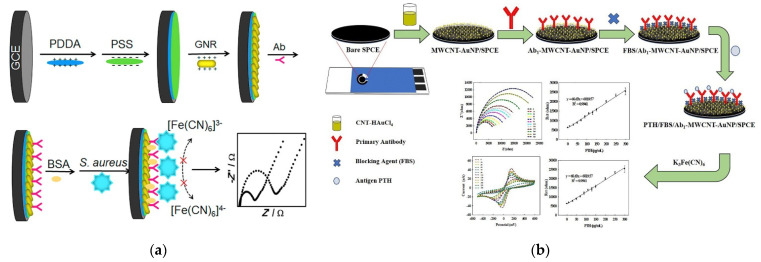
Examples of impedimetric immunosensors with non-covalently immobilized antibodies. (**a**) AuNR-based immunosensor. Antibodies were immobilized on the AuNR-modified electrode by physical adsorption. Reproduced with permission [73]. Copyright (2020) Elsevier. (**b**) MWCNT–AuNP nanocomposite-based immunosensor. The capture antibodies were immobilized on MWCNT–AuNP nanocomposite using physical adsorption. Reproduced with permission [74]. Copyright (2021) Elsevier.

Han et al. developed an impedimetric immunosensor using AuNR for the detection of *Staphylococcus aureus* [73]. They immobilized AuNRs onto an electrode using charge interaction. Before AuNR immobilization, positively charged poly-(diallyldimethylammonium chloride) and PSS were sequentially added to the electrode, and then positively charged AuNR was immobilized (Figure 5a). After AuNR immobilization, antibodies against *S. aureus* were immobilized using physical adsorption, and after bacterial binding, the signals were measured using EIS. The developed biosensor showed a 2.4 × 10^2^ CFU/mL LOD with a 1.8 × 10^3^–1.8 × 10^7^ CFU/mL linear range.

Malla et al. fabricated a PTH immunosensor based on nanocomposites of AuNPs and MWCNTs [74]. As shown in Figure 5b, the synthesis of AuNPs on MWCNTs and the modification of the electrode with MWCNT–AuNP nanocomposites were conducted in a single step using controlled-potential electrodeposition. After the modification of the electrode, the anti-PTH antibodies were immobilized using physical adsorption, followed by hormone treatment. Then, the signals were measured using both CV and EIS, and the LOD of each measurement was calculated to be 0.092 pg/mL and 0.033 pg/mL, respectively, with a linear range of 1–300 pg/mL.

### 4.2. Chemical Immobilization of Antibodies

Various impedimetric immunosensors based on covalently immobilized antibodies have been developed, such as that of Nawaz et al., who immobilized antibodies on electrografted proteins through covalent bonds for the diagnosis of dengue virus [75]. BSA was electrografted onto the electrode to induce antifouling properties and enhanced conductivity, and anti-NS1 antibodies were chemically immobilized using EDC/NHS chemistry. The formation of an immunocomplex was confirmed using EIS measurement, and the calculated LOD was 0.3 ng/mL with a 1–200 ng/mL linear range. Nessark et al. developed an impedance immunosensor by electrografting a polymer to detect IL-10 [76]. For the fabrication of the transducer, silicon dioxide and silicon nitride were sequentially deposited onto a silicon substrate using chemical vapor deposition. Then, the immunoaffinity layer was fabricated using silanization and polymerization. The electrode was silanized using SPy and modified with PPy. Then, the polymer-modified electrode was electrochemically modified with a carboxyl group. Antibodies were immobilized on the electrode through the carboxyl group using EDC/NHS. The fabricated immunosensor showed a sensitivity of 0.1128 pg/mL and an LOD of 0.347 pg/mL, with a detection range of 1–50 pg/mL.

Sadighbayan et al. and Aydın et al. fabricated various impedimetric immunosensors with chemically immobilized antibody layers based on an ITO transducer [77,78,79,80,81,82]. For IL-8 detection, antibodies were immobilized onto a PHA SAM layer using EDC/NHS chemistry (LOD: 7.5 fg/mL) and an epoxy group was placed on PGMA and the CB-functionalized electrode (LOD: 3.3 fg/mL) [77,78]. To detect tumor marker p53, a spin-coated CS–CB composite layer and glutaraldehyde cross-linking were used (LOD: 3 fg/mL) with an epoxy group on the PGMA spin electrode (LOD: 3.3 fg/mL) [79,80]. For CCR4 detection, an ITO electrode with acid-substituted PPy (PPy-COOH) was used to maximize the antibody binding sites, and antibodies were chemically bound using EDC/NHS (LOD: 6.4 fg/mL) [81]. Recently, a SARS-CoV-2 impedimetric immunosensor was developed using AuNPs [82]. The AuNPs were capped with a SAM layer to expose the carboxyl group, and the modified NPs were immobilized onto the ITO electrode. Then, the antibodies were immobilized through chemical bonds using EDC/NHS, and the fabricated biosensor showed an LOD of 0.577 fg/mL with a 0.002–100 pg/mL linear range.

**Figure 6:**
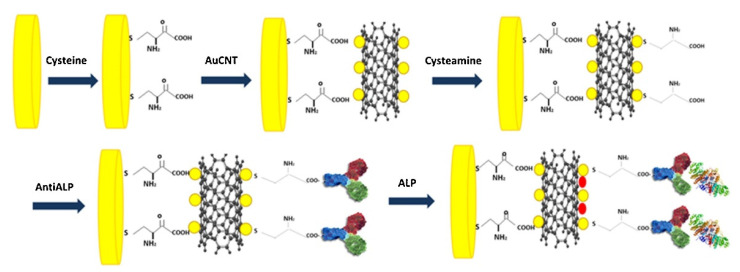
Example of impedimetric immunosensors with covalently immobilized antibodies. MWCNT–AuNP-based immunosensor was fabricated by the immobilization of antibodies on carboxyl-functionalized MWCNT–AuNP using EDC/NHS chemistry. Reproduced with permission [83]. Copyright (2018) Elsevier.

Carbon-based materials, such as MWCNTs, have also been used to fabricate impedimetric immunosensors. Simão et al. fabricated an MWCNT–AuNP-based immunoaffinity layer to detect ALP [83]. As shown in Figure 6, the MWCNT–AuNPs on the electrode were functionalized with carboxyl groups using cysteamine, and the antibodies were covalently immobilized using EDC/NHS chemistry. After analyte binding, the signal was measured using EIS, and the LOD was calculated to be 0.25 IU/L with a 0.5–600 IU/L detection range. Vasantham et al. fabricated a paper-based immunosensor utilizing carboxyl-functionalized MWCNTs to detect cTnI [84]. After covalently immobilizing the capture antibodies, the EIS measurement showed a 0.05 ng/mL LOD and 1.85 mΩ/ng/mL sensitivity, with a 0.05–50 ng/mL detection range.

## 5. ECL Immunosensors

### 5.1. Physical Adsorption of Antibodies

ECL, also known as electrogenerated chemiluminescence, is a type of chemiluminescence in which luminophores generate electronically excited states by electron transfer on the electrode surface [85,86]. Consequently, the ECL technique does not require any external light source for excitation; therefore, it not only simplifies the measuring system, but it also reduces the background noise signal from the scattered light source or the autofluorescence of the analyte compared with a chemiluminescence system [87,88,89]. In addition, the ECL technique has become a powerful analytical method owing to its high sensitivity and stability [90,91,92,93]. Thus, ECL has been used for various applications, such as clinical diagnosis, environmental monitoring, and food monitoring [94,95,96]. However, ECL has the disadvantage of requiring instruments, including emission detection systems [97,98].

**Figure 7:**
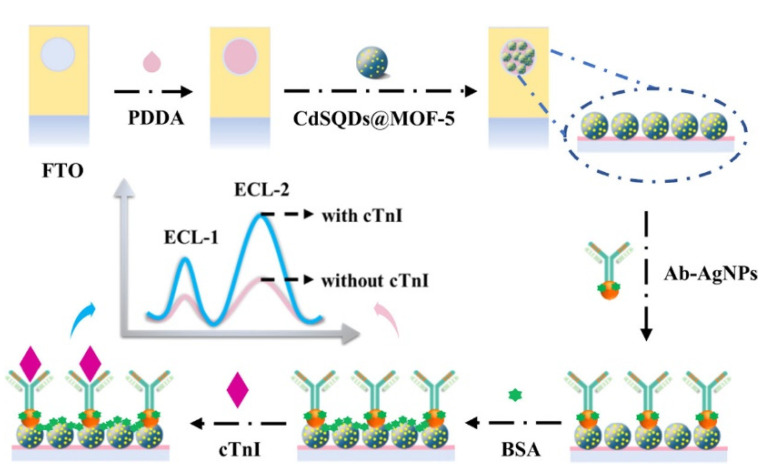
Examples of ECL immunosensors with physically adsorbed capture antibodies. Antibodies were immobilized on AgNPs to form an immunoaffinity layer. Reproduced with permission [100]. Copyright (2020) American Chemical Society.

Liu et al. used AgNPs to form an immunoaffinity layer to detect cyclin D1 [99]. The electrode was modified with a PDA–AgNP composite, and capture antibodies were immobilized using physical adsorption. For signal generation, detection antibodies conjugated with AuNPs and Bi_2_S_3_ QD-based nanoprobes were used. After forming the sandwich immunocomplex, the ECL measurement showed a 6.34 fg/mL LOD with a 10 fg/mL–1 μg/mL linear range. Du et al. fabricated an ECL immunosensor with CdS QDs and AgNPs for the detection of cTn-I [100]. In that study, the luminophore was firstly immobilized on an immunoaffinity layer (Figure 7). The synthesized nanoluminophore, MOF-5-encapsulated CdS QD (CdSQD@MOF-5), was coated with a PDDA-modified electrode. Then, AgNPs-conjugated antibodies were immobilized to form an immunoaffinity layer. cTnI was quantified using an ECL-based measurement and the result showed a 5.01 fg/mL LOD with a 0.01–1000 pg/mL linear range.

Various ECL immunosensors have been developed using an AuNP-based immunoaffinity layer with physically adsorbed capture antibodies. Studies by Wang et al. and Yang et al. from the Yuan group developed ECL immunosensors using physically immobilized antibodies on electrodeposited AuNPs [101,102]. They used a compound consisting of RU derivative (RUD), PEI, and ABEI as luminophores to increase the ECL signal using ECL resonance energy transfer to detect Col IV [101]. This immunosensor showed a 0.17 pg/mL LOD with a 0.5 pg/mL–7.2 ng/mL detection range. In another study, they used PFO dots instead of RUD to fabricate a KIM-1 immunosensor [102]. The ECL emitter, an ABEI-PER-PFO dot, was combined with rGO-PtNP for conjugation with detection antibodies. After forming the sandwich immunocomplex, the LOD and linear range were calculated to be 16.7 fg/mL and 50 fg/mL–1 ng/mL, respectively, using ECL measurement. Zheng et al. fabricated a CEA immunosensor using an AuNP–Ab immunoaffinity layer [103]. Antibodies were immobilized onto the AuNP-modified electrode using physical adsorption. To generate ECL signals, compounds consisting of PDDA-rGO and ZnSe/ZnS QDs were conjugated with detection antibodies. CEA levels were then measured using ECL, and the fabricated immunosensor showed an LOD of 0.029 pg/mL with a linear range of 0.0001–100 ng/mL. Lian et al. developed a label-free ECL immunosensor using Au–Co alloy NPs (Au–Co NPs) for the detection of LDL and oxidized LDL (ox-LDL) [104]. Au–Co NPs were immobilized onto an APTMS-silanized electrode, and each antibody against LDL and ox-LDL was immobilized. After antigen binding and ECL measurement, the calculated LODs were 0.256 pg/mL and 0.330 pg/mL, and the calculated linear ranges were 0.420–100 pg/mL and 0.500–60.0 pg/mL, for LDL and ox-LDL, respectively.

Gold nanomaterials have also been used with carbon-based materials for the fabrication of immunoaffinity layers. Qin et al. modified an electrode with a nanocomposite consisting of Ru–SiO_2_, AuNP, and rGO (rGO@AuNP@RU-SiO_2_) to detect AFP using ECL [105]. After sequential antibody and antigen treatment, the ECL measurement showed a 0.03 pg/mL LOD, with a 0.0001–100 ng/mL linear range. Khan et al. fabricated a label-free ECL immunosensor using Ce_2_Sn_2_O_7_–AuNP nanocubes for the detection of CEA [106]. For the fabrication of the immunosensor, nanocubes and capture antibodies were sequentially treated. After antigen treatment, the ECL signal was measured, and the developed immunosensor presented a linear range of 0.001–70 ng/mL and an LOD of 0.53 pg/mL.

### 5.2. Chemical Immobilization of Antibodies

Covalent immobilization is also frequently used for the formation of immunoaffinity layers in ECL biosensors. Babamiri et al. fabricated an HBsAg immunosensor using magnetite NPs [107]. Antibodies against HBsAg were covalently immobilized onto carboxyl-modified Fe_3_O_4_ NPs via EDC/NHS chemistry. For the amplification of the ECL signal, CdTe/CdS QDs were used to form nanoclusters with PAMAM dendrimers, and detection antibodies were immobilized onto the fabricated nanoclusters. After the formation of the sandwich immunocomplex, HBsAg was quantified using ECL measurement, and the results showed a 0.80 fg/mL LOD and a 3 fg/mL–0.3 ng/mL linear range.

Fang et al. developed an ECL immunosensor using a carbon-based nanomaterial, g-C_3_N_4_, to detect HE4 [108]. They synthesized carboxyl-wrapped g-C_3_N_4_ and made it into a composite with mesoporous silica (g-C_3_N_4_@SiO_2_). The capture antibodies were immobilized via the carboxyl group of g-C_3_N_4_ by EDC/NHS. For signal amplification, detection antibodies were chemically immobilized onto the carbon nanohorn–polymer dot composites. The fabricated ECL immunosensor showed a 3.3 × 10^−6^ ng/mL LOD with a 1.0 × 10^−5^–10 ng/mL linear range. Ding et al. used another carbon-based nanomaterial, MWCNT, to detect 5hmC [109]. For the immobilization of antibodies, the electrode was modified with MWCNTs, and capture antibodies were chemically immobilized using EDC/NHS chemistry. After analyte treatment, detection antibodies conjugated with PAMAM–silver nanoclusters and nitrogen-doped graphene nanocomposites were used as ECL probes, and signals were measured using ECL. The calculated LOD and linear range were 2.47 pM and 10 pM–30 nM, respectively.

**Figure 8:**
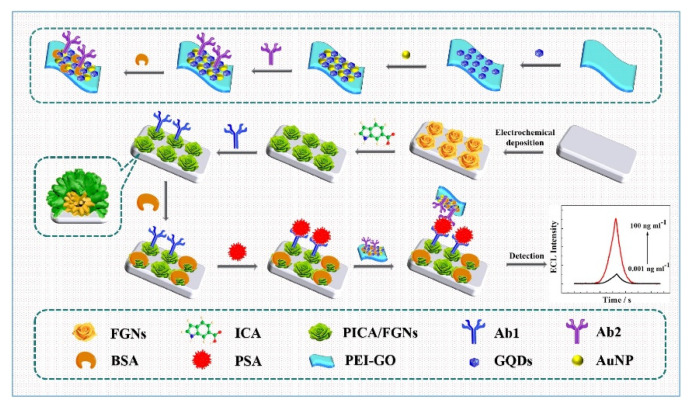
Examples of ECL immunosensors with covalently immobilized capture antibodies. The capture antibodies were chemically immobilized on a PICA–AuNP nanocomposite-modified electrode using EDC/NHS. Reproduced with permission [110]. Copyright (2020) Elsevier.

Yang et al. used AuNPs for the detection of PSA [110]. In that study, PICA was electropolymerized onto electrodeposited flower-like AuNPs to form PICA–AuNP nanocomposites (Figure 8). The capture antibodies were then chemically immobilized using EDC/NHS. For the ECL probe, detection antibodies were immobilized onto GQDs and AuNP-immobilized PEI-modified GO. The fabricated ECL immunosensor showed a 0.44 pg/mL LOD with a 0.001–10 ng/mL linear range.

## 6. Conclusions and Perspectives

In this review, various electrochemical, voltammetric, amperometric, impedimetric, and ECL immunosensors have been introduced, as summarized in Table 1 and further discussed. In particular, we focused on the immobilization of antibodies for the fabrication of immunoaffinity layers, by dividing them into those immobilized using physical adsorption and those using chemical bonding. Generally, physical adsorption methods use not only simple immersion, but also attractive materials known to have an affinity for antibodies, such as AuNPs. To improve the performance of immunosensors, enhancing the effectiveness of antibodies, especially by increasing exposed antigen binding sites, is a key technique. Therefore, advanced antibody adsorption strategies, such as introducing the orientation control of antibodies, scFv, or nanobodies, would be advantageous.

For covalent immobilization, electrodes or binding materials are modified with functional groups such as carboxyl, amine, and hydroxyl using various chemistry-based techniques, including SAM and silanization. Among them, EDC/NHS chemistry is one of the most frequently used methods for binding the amine groups of antibodies to carboxylated surfaces. In addition, silanization methods are frequently used for hydroxyl surfaces, such as ITO electrodes. Covalent immobilization can irreversibly immobilize antibodies to form a robust immunoaffinity layer. However, most chemical reactions require the linking and blocking of the functional groups in antibodies, which can affect the binding activity. In addition, blocking the Fab region, including the antigen-binding paratope, would limit the performance of immunosensors. Thus, Fc-binding strategies can improve the performance of electrochemical immunosensors by enhancing the functionalization of electrodes with high density and efficiency.

Electrochemical biosensors have been developed in the past few decades, and remain active research areas in analytical chemistry that offer advantages such as small sample volume, cost-effectiveness, simplicity, high sensitivity, reproducibility, and selectivity. For electrochemical immunosensors, antibodies are used for the recognition and specific binding of analytes onto electrodes, and the labeling of bound target analytes with signal generating or amplifying molecules. In electrochemical biosensors, various nanomaterials are introduced into the electrodes or labeling molecules for various reasons, such as increasing conductivity, electron transfer, signal generation, and amplification. Therefore, the synthesis of highly effective nanomaterials would increase the performance of electrochemical biosensors. In conclusion, strategies for introducing highly effective nanomaterials or their composite conjugated antibodies with high-density antigen-binding sites need to be assessed in future studies.

## Figures and Tables

**Table 1:** Examples of electrochemical immunosensors.

Methods	Immunoaffinity Layer	Analyte	LOD	Detection Range	Ref.
DPV	GO/tryamine/Ab ^1^	CRP	1.25 µg/L (LOQ)	1.09–100 µg/L	[30]
	AuNP/Ab	PSA	0.12 pg/mL	0.0005–10.00 ng/mL	[33]
	Graphene/PPD/AuNP/Ab	NSE	0.3 ng/mL	1–1000 ng/mL	[37]
	CoS_2_-graphene/AuNP/Ab	CA15-3	0.03 U/mL	0.1–150 U/mL	[38]
	Graphene/MOFs/Ab	NMP22	1.7 pg/mL	0.005–20 ng/mL	[39]
	MWCNT/PANI/AuNP/Ab	PSA	0.5 pg/mL	1.66 ag/mL–1.3 ng/mL	[40]
	APTMS-Fe_3_O_4_/Ab	HER2	20 fg/mL	5 ×10^−4^–50 ng/mL	[43]
	GO–COOH/Ab	Mucin1	0.04 U/mL	0.1–2 U/mL	[44]
	GO–CS/Ab	CysC	0.0078 mg/mL	1–10 mg/mL	[45]
	Graphene/Antigen	HSP70	0.02 ng/mL	0.0448–100 ng/mL	[47]
DPV SWV	MWCNT/AuNP/Ab	PTH	886 fg/mL 65 fg/mL	1–300 pg/mL	[41]
SWV	AF-GO/PEI/Ab	CysC	0.03 ng/mL	0.1–1000 ng/mL	[31]
	CS/AuNP/Ab	PSA	0.001 ng/mL	1–18 ng/mL	[34]
	PANI/AuNP/Ab	PSA	1.25 fg/mL	0.01–100 ng/mL	[35]
	CS-MWCNT-Fe_3_O_4_/Ab	CA19-9	0.163 pg/mL	0.001–100 ng/mL	[46]
SWV Amperometry	Au/Ab	procalcitonin	82.6 fg/mL 95.4 fg/mL	0.0001–50 ng/mL	[36]
CV	Avidin/biotinylated Ab	MMP-9 Apo-A4	0.21 ng/mL 6.6 ng/mL	0.4–100 ng/mL 10–100 ng/mL	[48]
Amperometry	Ab	MPT64	0.43 ng/mL	0.3–50 ng/mL 50–1000 ng/mL	[53]
	N-GQD/AuPdCu/Ab	HBsAg	3.3 fg/mL	10 fg/mL–50 ng/mL	[54]
	PtAg/Ab	CEA AFP	0.0005 ng/mL 0.001 ng/mL	0.001–40 ng/mL 0.005–100 ng/mL	[55]
	AuNP/Ab	AFP	6.7 fg/mL	20 fg/mL–100 ng/mL	[56]
	AuNP/Ab	CEA	6.7 fg/mL	20 fg/mL–200 ng/mL	[57]
	g-C_3_N_4_/AuNP/Ab	galectin-3	25.0 fg/mL	0.0001–20.0 ng/mL	[58]
	TiO_2_-rGO/AuNR@Pd/Ab	HE4Ag	13.33 fM	40 fM–60 nM	[59]
	pPPA/Ab	Endoglin	140 pg/mL	0.18–20 ng/mL	[60]
	Fe_3_O_4_@TMU-21/MWCNT/Ab	HER2	0.3 pg/mL	1.0 pg/mL–100 ng/mL	[61]
	AuNP/Ab	EV71	0.01 ng/mL	0.1–600 ng/mL	[62]
	AuNP-PAMAM/Ab	tau	1.7 pg/mL	6–5000 pg/mL	[63]
EIS	PDDA/PSS/AuNR/Ab	*S. aureus*	2.4 × 10^2^ CFU/mL	1.8 × 10^3^–1.8 × 10^7^ CFU/mL	[73]
	BSA/Ab	NS1	0.3 ng/mL	1–200 ng/mL	[75]
	SPy-PPy/Ab	IL-10	0.347 pg/mL	1–50 pg/mL	[76]
	PHA/Ab	IL-8	7.5 fg/mL	0.025–3 pg/mL	[77]
	PGMA-CB/Ab	IL-8	3.3 fg/mL	0.01–3 pg/mL	[78]
	Chitosan-CB	P53	3 fg/mL	0.01–2 pg/mL	[79]
	PGMA/Ab	P53	7 fg/mL	0.02–4 pg/mL	[80]
	PPy-COOH/Ab	CCR4	6.4 fg/mL	0.02–8 pg/mL	[81]
	AuNP-SAM/Ab	SARS-CoV-2	0.577 fg/mL	0.002–100 pg/mL	[82]
	MWCNT–AuNP/Ab	ALP	0.25 IU/L	0.5–600 IU/L	[83]
	MWCNT-COOH/Ab	cTnI	0.05 ng/mL	0.05–50 ng/mL	[84]
EIS CV	MWCNT–AuNP/Ab	PTH	0.033 pg/mL 0.092 pg/mL	1–300 pg/mL	[74]
ECL	PDA-AgNP/Ab	Cyclin D1	6.34 fg/mL	10 fg/mL–1 μg/mL	[99]
	CdSQD@MOF-5/AgNP-Ab	cTnI	5.01 fg/mL	0.01–1000 pg/mL	[100]
	AuNP/Ab	Col IV	0.17 pg/mL	0.5 pg/mL–7.2 ng/mL	[101]
	AuNP/Ab	KIM-1	16.7 fg/mL	50 fg/mL–1 ng/mL	[102]
	AuNP/Ab	CEA	0.029 pg/mL	0.0001–100 ng/mL	[103]
	APTMS/Au–Co NPs/Ab	LDL Ox-LDL	0.256 pg/mL 0.330 pg/mL	0.420–100 pg/mL 0.5–60 pg/mL	[104]
	rGO@AuNP@RU-SiO_2_/Ab	AFP	0.03 pg/mL	0.0001–100 ng/mL	[105]
	Ce_2_Sn_2_O_7_-AuNP/Ab	CEA	0.53 pg/mL	0.001–70 ng/mL	[106]
	Fe_3_O_4_/Ab	HBsAg	0.80 fg/mL	3 fg/mL–0.3 ng/mL	[107]
	g-C_3_N_4_@SiO_2_/Ab	HE4	3.3×× 10^−6^ ng/mL	1.0×× 10^−5^–10 ng/mL	[108]
	MWCNT/Ab	5hmC	2.47 pM	10 pM–30 nM	[109]
	AuNP/PICA/Ab	PSA	0.44 pg/mL	0.001–100 ng/mL	[110]

^1^ Antibodies.

## Data Availability

Not applicable.

## Supplementary Materials

The following are available online at https://www.mdpi.com/article/10.3390/bios11100360/s1. Table S1: Abbreviations used in this work.
