# Supplementary material for: Recent Progress in Electrochemical Immunosensors
Source: Biosensors (Basel). 2021 Sep 29;11(10):360. doi: 10.3390/bios11100360 (PMC8533705; doi:10.3390/bios11100360)
Supplement: Supplementary file 1 [file biosensors-11-00360-s001.zip › biosensors-1381692-supplementary.pdf]

biosensors

MDPI

Recent Progress in Electrochemical Immunosensors

Jeeyoung Kim [1,2,3] and Min Park [1,2,3,*]

[1] Major in Materials Science and Engineering, Hallym University, Chuncheon 24252, Gangwon-do, Korea; jyoung@hallym.ac.kr
[2] Cooperative Course of Nano-Medical Device Engineering, Hallym University, Chuncheon 24252, Gangwon-do, Korea
[3] Integrative Materials Research Institute, Hallym University, Chuncheon 24252, Gangwon-do, Korea
* Correspondence: minpark@hallym.ac.kr

Table S1. Abbreviations used in this work.

Abbreviation	Full name	Abbreviation	Full name
EIS	electrochemiluminescent	CMOS	complementary metal oxide semiconductor
LSV	linear sweep voltammetry	DPV	differential pulse voltammetry
SWV	square wave voltammetry	CV	cyclic voltammetry
CRP	C-reactive protein	GO	graphene oxide
LOQ	limit of quantification	CysC	cystatin C
AF	aminoferrocene	PEI	polyethyleneimine
LOD	limit of detection	AuNP	gold nanoparticle
PSA	prostate-specific antigen	HP5	hydroxyl pillar arene
CS	chitosan	HRP	horseradish peroxidase
PANI	polyaniline	PDA	Polydopamine
NSE	neuron-specific enolase	PPD	poly para-phenylenediamine
CA15-3	carbohydrate antigen 15-3	NMP22	nuclear matrix protein 22
MOF	metal organic framework	PtNP	platinum nanoparticle
CNT	carbon nanotube	MWCNT	multi-welled carbon nanotube
PTH	parathyroid hormone	HER2	epidermal growth factor receptor 2
APTMS	3-aminopropyltrimethoxysilane	EDC	N-(3-dimethylaminopropyl)-N′-ethylcarbodiimide hydrochloride
NHS	N-hydroxysuccinimide	HSP70	heat shock protein 70
ALP	alkaline phosphatase	MMP-9	matrix metallopeptidase-9
Apo-A4	apolipoprotein-A4	MPT64	*Mycobacterium tuberculosis* protein 64
TMB	*3,3′-5,5′*-tetramethylbenzidine	HBsAg	hepatitis B surface antigen
N-GQD	nitrogen-doped graphene quantum dot	CEA	carcinoembryonic antigen
AFP	alpha-fetoprotein	AgNP	silver nanoparticle
rGO	reduced graphene oxide	g-C_3N_4	graphitic carbon nitride nanosheets
AuNR	gold nanorod	HE4Ag	human epididymis specific protein 4 antigen
pPPA	poly(pyrrolepropionic) acid	EV71	human enterovirus 71
SAM	self-assembly monolayers	PAMAM	poly(amidoamine)
EIS	impedance spectroscopy	PSS	poly-(styrenesulfonate)
BSA	bovine serum albumin	NS1	non-structural 1
IL	interleukin	SPy	pyrrole–silane
PPy	polypyrrole	ITO	indium tin oxide
PHA	phosphonohexanoic acid	PGMA	poly(glycidyl methacrylate)
CB	carbon black	SARS-CoV-2	severe acute respiratory syndrome coronavirus 2
CCR4	C-C motif chemokine receptor 4	cTnI	cardiac troponin I

QD	quantum dot	PDDA	poly(diallyldimethylammonium chloride)
RU	tris(bipyridine) ruthenium(II)	ABEI	N-(aminobutyl)-N-(ethylisoluminol)
Col IV	collagen type IV	PFO	poly(9,9-dioctylfluorenyl-2,7-diyl)
KIM-1	kidney injury molecule-1	LDL	low-density lipoprotein
HE4	human epididymis protein 4	5hmC	5-hydroxymethylcytosine
PICA	poly(indole-6-carboxylic acid)	scFv	single chain variable fragment
